# P-1052. Identifying Facility and Specimen Type Associated with Screening-to-Clinical *Candida auris* Patients in the United States, 2013–2022

**DOI:** 10.1093/ofid/ofae631.1241

**Published:** 2025-01-29

**Authors:** Anna D Baker, Sophie Jones, Malavika Rajeev, Meghan Lyman

**Affiliations:** CDC, Atlanta, Georgia; CDC, Atlanta, Georgia; Centers for Disease Control, Atlanta, Georgia; Centers for Disease Control and Prevention, Atlanta, Georgia

## Abstract

**Background:**

*Candida auris* (*C. auris*) is an emerging healthcare-associated fungal pathogen associated with high mortality and antifungal resistance. Targeted screening based on patient risk factors and facility type is important for identifying colonized patients and implementing recommended infection prevention and control (IPC) precautions. Understanding the epidemiology of *C. auris* colonized patients who subsequently have positive clinical specimens (screening-to-clinical patients (STC)) can help us understand the clinical course and impact of colonization.

**Methods:**

Using national case surveillance data from 34 states and Washington DC, we described *C. auris* adult patients, as defined by Council of State and Territorial Epidemiologists, during 2013–2022. We analyzed facility and specimen source for STC patients and compared with screening or clinical only patients using chi-squared tests.

**Results:**

Among 13,341 screening patients, 1,151 (9%) were STC. STC patients were more likely than screening only patients to be screened at long-term acute-care hospitals (LTACH) (48% vs 40%; p< 0.001) and were more likely than clinical only patients to have positive clinical specimens taken at LTACHs (35% vs 10%; p< 0.001). While blood was the most frequent clinical specimen source for STC and clinical only patients (39% and 35% respectively), respiratory specimens were more likely to be taken from STC patients than clinical only patients (12% vs 8%; p< 0.001). The median time between STC specimen collection was 47 days [interquartile range (IQR) 19-111] and was shorter for patients in LTACHs (median 37 days [IQR 17–78]) than ACHs (median 53 days [IQR 21–116]).

**Conclusion:**

About half of STC patients were screened at LTACHs and over half of STC clinical specimens were collected at LTACHs. While blood specimens were most frequent clinical specimen source among STC, respiratory sources were more likely to be taken from STC patients than clinical only patients. As high-risk respiratory devices (e.g., mechanical ventilation) are associated with invasive infections, our findings emphasize the importance of timely diagnosis and monitoring STC patients with clinical respiratory sources in LTACHs to reduce risk of invasive infection.

**Disclosures:**

**All Authors**: No reported disclosures

